# Supplementary material for: The Effects of Increasing Fruit and Vegetable Intake in Children with Asthma on the Modulation of Innate Immune Responses
Source: Nutrients. 2022 Jul 27;14(15):3087. doi: 10.3390/nu14153087 (PMC9370535; doi:10.3390/nu14153087)

Figure S1- The Consolidated Standards of Reporting Trials diagram for the per-protocol analysis of the randomized controlled trial investigating the effect of a high fruit and vegetable intervention in children with asthma.

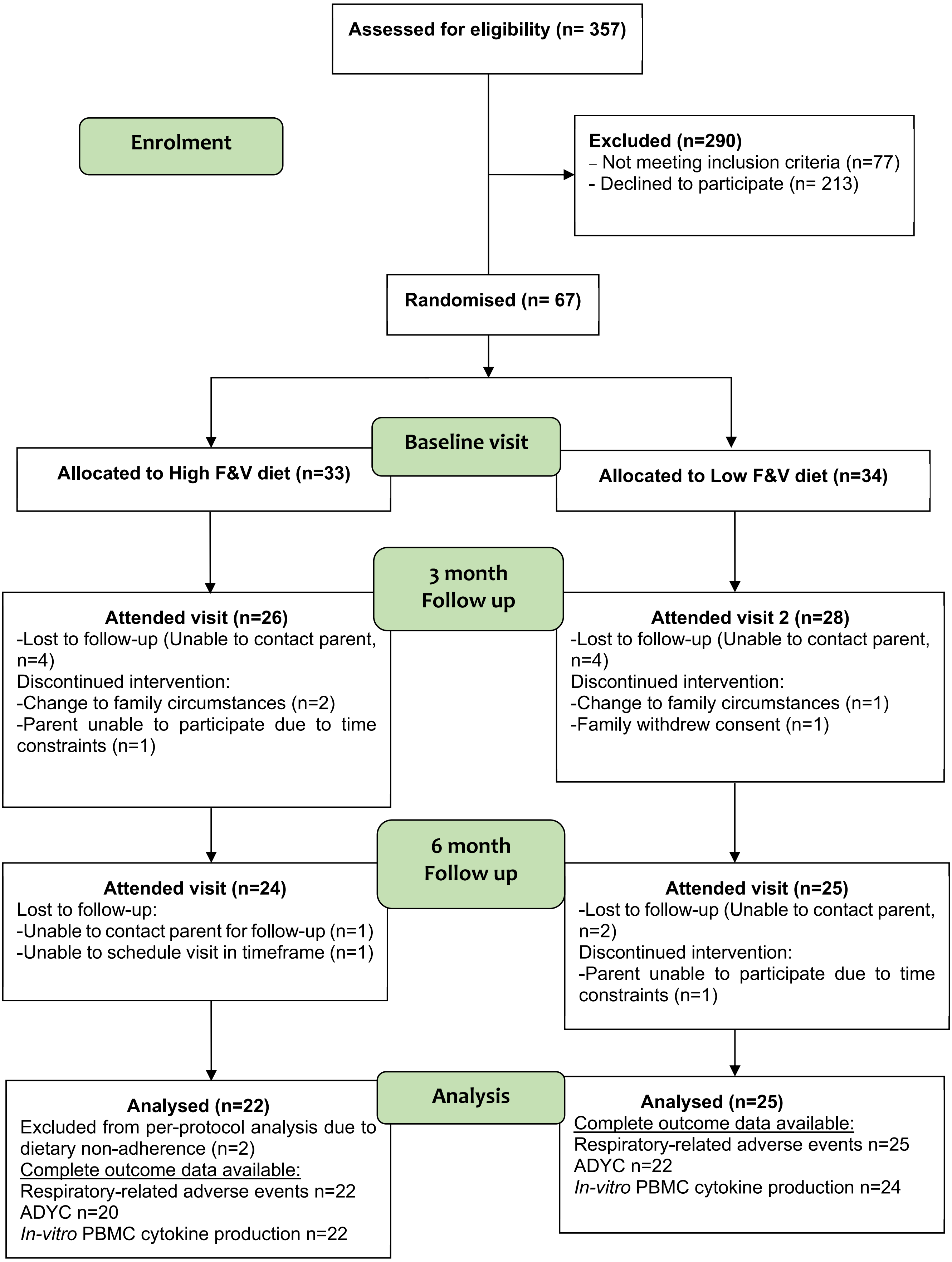

Supplement: Supplementary file 1 [file nutrients-14-03087-s001.zip › Figure S1.pdf]
