# Supplementary material for: The Effects of Increasing Fruit and Vegetable Intake in Children with Asthma on the Modulation of Innate Immune Responses
Source: Nutrients. 2022 Jul 27;14(15):3087. doi: 10.3390/nu14153087 (PMC9370535; doi:10.3390/nu14153087)
Supplement: Supplementary file 1 [file nutrients-14-03087-s001.zip › Supplementary File S1.pdf]

Supplementary Materials

METHODS

Questionnaire during an asthma exacerbation or upper respiratory tract infection (URTI) (episode)

At the time of a suspected URTI or asthma exacerbation, the parent/guardian was asked to complete the validated Asthma Diary for Young Children (ADYC)[2]. Events recoded in the ADYC were considered exacerbations and analysed if they were for 2 or more consecutive days which contained no more than 1 missing or cannot answer item per day, with at least 2 of 3 symptoms of cough, difficulty breathing or wheezing, scoring 2 or more. Exacerbation event duration in days was calculated as the number of days from the first day with 2 or more symptoms to last day to one or more symptoms. The cumulative ADYC score was the sum of the daily score for the duration of each event. Exacerbations with missing or incomplete ADYCs were discarded.

Nasal swab collection

At baseline, parents were provided with several nasal swabs, 1 ml tube containing Universal Transport Medium (UTM) and a cooler-bag for later transport of any samples collected between visits. Instructions on sample collection were provided at the first visit by research staff who also demonstrated the technique by collecting the initial nasal swab specimen from the child. At the onset of an asthma exacerbation or a suspected URTI (that is, no later than day one or two of symptoms), a sample of nasal secretions was collected by parents by gently inserting a flocked mid-turbinate paediatric swab in one nostril and gently rotating, before removing and placing the swab in a UTM tube. Parents were asked to store the sample(s) in the freezer until their next visit (at 3 months or 6 months), at which time they transported the frozen samples to research personnel in the provided cooler-bag.

PBMC isolation and culture

PBMC isolation and culture

PBMCs were resuspended in Roswell Park Memorial Institute media (Invitrogen, Australia Pty Limited), 5% foetal bovine serum (Invitrogen, Australia Pty Limited). Cell viability was evaluated by trypan blue staining, which differentiates live cells from dead cells based on cellular necrosis. Cell counts were performed. PBMCs were then

adjusted to a concentration of 1.0 x 10^6 viable cells per well in 24-well plates (sterile, tissue culture grade, Australia) and cultured with medium alone (control) or with RV-1β at a final concentration of 20 multiplicity of infection, or HDM (Greer Laboratories, USA) at final concentration of 100ug/mL[3] or LPS (Sigma-Aldrich, USA) at a final concentration of 100ng/mL[4] for a total of 48h, at 33°C and 5% CO_2[5]. The media was not changed during this period. Cellular suspensions were centrifuged at 550Xg for 10 min and supernatants stored at -80°C for subsequent analyses. Experimental protocol and procedures were approved by the University of Newcastle Biosafety Committee (approval number R5/2017).

Cytokine assays

The concentrations of IFN-γ, IL-1β and IL-6 in the culture supernatants were analysed using bead-based multiplex assay (BD Bioscience, Sydney, Australia). The assay range was 10.0-2500 pg/ml. The minimum detection limit of this assay was 1.8 pg/ml. IFN-λ and IL-5 concentrations in the culture supernatants were measured using a high-sensitivity commercial ELISA assay (R&D Systems, Sydney, Australia), as per the manufacturer's recommendations. The assay range was 50-4000 pg/ml. The minimal detectable dose was ≤10 pg/ml. All samples were tested in duplicates and the %CV between duplicate samples was accepted when <10%.

Respiratory viruses in nasal samples

Viral RNA was extracted from 140 µL of each nasal sample using the QIAamp Viral RNA Mini Kit (Qiagen, Australia) following the manufacture's instructions[6]. To synthesize cDNA, reverse transcription (RT) was performed using the High Capacity cDNA Reverse Transcription Kit (Applied Biosystems, Australia). One µL of cDNA was then added to 11.5 µL qPCR reaction containing 2 x Taqman PCR Master Mix (Qiagen, Australia), virus-specific primers and probes. The PCR detected RV, coronaviruses (CoV), influenza viruses A and B, and respiratory syncytial viruses (RSV) A and B. Negative and positive template controls were included with every run to validate PCR. All samples were analysed in duplicate and considered positive only if virus was detected in both samples

References

1. National Health and Medical Research Council. Australian Dietary Guidelines. Canberra: National Health and Medical Research Council, 2013.
2. Ducharme F, Mendelson M, Klassen TP, et al. The Preschool Asthma Diary (PAD): A validated instrument for day-to-day monitoring of asthma severity in young children. Pediatric Academic Societies 2007:6310.6. (http://www.abstracts2view.com/pasall).
3. Mishra A, Brown AL, Yao X, et al. Dendritic cells induce Th2-mediated airway inflammatory responses to house dust mite via DNA-dependent protein kinase. Nature communications 2015;6:6224. (In eng). DOI: 10.1038/ncomms7224.
4. Plevin RE, Knoll M, McKay M, Arbabi S, Cuschieri J. The Role of Lipopolysaccharide Structure in Monocyte Activation and Cytokine Secretion. Shock 2016;45(1):22-7. (In eng). DOI: 10.1097/shk.0000000000000470.
5. Forbes RL, Gibson PG, Murphy VE, Wark PA. Impaired type I and III interferon response to rhinovirus infection during pregnancy and asthma. Thorax 2012;67(3):209-14. (In eng). DOI: 10.1136/thoraxjnl-2011-200708.
6. QIAGEN Viral RNA Mini Kit handbook – available online at: https://www.qiagen.com/au/resources/resourcedetail?id=c80685c0-4103-49ea-aa72-8989420e3018&lang=en.
